# Acupuncture for enhancing early recovery of bowel function in cancer: Protocol for a systematic review

**DOI:** 10.1097/MD.0000000000006644

**Published:** 2017-04-28

**Authors:** Yi-Hua Liu, Yang Ye, Jia-Bin Zheng, Xue-Qian Wang, Ying Zhang, Hong-Sheng Lin

**Affiliations:** a Graduate School of Beijing University of Chinese Medicine; b Department of Oncology, Guang’anmen Hospital, China Academy of Chinese Medical Sciences, Beijing, China.

**Keywords:** acupuncture, bowel function, cancer, POI, protocol, systematic review

## Abstract

**Background:**

Cancer patients undergoing surgical procedure often suffer from bowel dysfunction and postoperative ileus (POI). Cancer management for early recovery of bowel function is still a challenging topic. Acupuncture has been commonly used in a variety of gastrointestinal diseases. The aim of this study is to evaluate the effects of acupuncture therapy to reduce the duration of POI and enhance bowel function in cancer patients.

**Methods:**

We will systematically screen all randomized controlled trials (RCTs) published through electronically and hand searching. The following search engines including Medline, EMBASE, Cochrane CENTRAL, the Cumulative Index to Nursing and Allied Health Literature, Allied and Complementary Medicine Database, the Chinese Biomedical Literature Database, the China National Knowledge Infrastructure, VIP Information, Wanfang Data, one Japanese database (Japan Science and Technology Information Aggregator, Electronic) and 2 Korean Medical Databases (Korean Studies Information, and Data Base Periodical Information Academic) will be retrieved. Supplementary sources will be searched including gray literature, conference proceedings, and potential identified publications. Two reviewers will independently conduct the trial inclusion, data extraction and assess the quality of studies. The time to first passing flatus and time to first bowel motion will be assessed as the primary outcomes. Adverse effects, time to first bowel sound, visual analog scale (VAS) pain score, hospital stay, and postoperative analgesic requirement will be measured as secondary outcomes. Methodological quality will be evaluated according to the Cochrane risk of bias. Details of interventions will be assessed by the Standards for Reporting Interventions in Controlled Trials of Acupuncture (STRICTA) checklist. All analyses will be applied by RevMan (version 5.3) and StataSE (version 12).

**Ethics and Dissemination:**

This systematic review will provide up-to-date information on acupuncture therapy for early recovery of bowel function in cancer patients. This review does not require ethical approval and will be reported in a peer-reviewed journal and presented at a relevant conference.

**Trial Registration Number:**

PROSPERO CRD42016049633.

## Abstract 2

Supplemental Digital Content is available in the text

## 1 Introduction

Bowel dysfunction has been found to be closely related to worse postoperative quality of life, which is regarded as a major outcome measure in surgical oncology.^[1,2]^ A temporary impairment of bowel motility, known as postoperative ileus (POI), is expected after any major surgical procedure, including cancer surgery.^[3]^ The symptoms of POI include abdominal distension, nausea, vomiting, inability to tolerate an oral diet or a mix of them.^[4]^ Risk factors for POI include long time of surgery, hemorrhage, and extensive manipulations of abdominal cavity.^[5]^ Since these conditions frequently occur in cancer patients, it can be expected that cancer patients would have more risk of developing POI. In addition to the discomfort experience, POI is associated with delayed patient recovery, prolonged length of hospital stay, and increased healthcare costs.^[6]^ An epidemiological study found that POI has been variably reported from 10% to 32% of patients who have undergone any major surgery.^[7]^ The total estimated annual economic burden of POI is over $750 million in the United States.^[8]^

Since POI is one of the major causes for the delayed recovery of bowel function following cancer resection,^[9]^ pharmacological and nonpharmacological therapies have been directed toward alleviating POI.^[8]^ Nonpharmacological techniques such as laparoscopic surgery and fast-track recovery programmes are primarily aimed at smaller incisions, reduced pain, and improved perioperative care management; pharmacological agents such as cyclooxygenase 2 (COX-2) inhibitors, ghrelin agonists, and opioid agonists focus on decreasing inflammation or acting on μ-opioid-receptor.^[10]^ Despite the limited efficacy of these interventions, patients often switch to integrative therapies due to the side effects and economic burden.

For thousands of years, acupuncture has been widely practiced in China as an effective treatment option for the management of gastrointestinal diseases.^[11]^ According to the theory of traditional Chinese medicine, surgery causes *Qi* stagnation and blood stasis syndrome, breaks the balanced state of human body, and eventually leads to bowel dysfunction.^[12]^ Acupuncture is believed to have the function of regulating the energy (*Qi)* flow and removing blood stasis by needling at specific acupuncture points. Moreover, acupuncture has been widely practiced as an effective treatment option for cancer symptoms, such as cancer pain, cancer-related hot flashes, and chemotherapy-induced nausea/vomiting.^[11,13]^ However, whether acupuncture has a definite therapeutic effect on enhancing bowel function in cancer patients remains controversial. Studies have reported conflicting results addressed to this issue. Previous systematic reviews found limited results supporting acupuncture as an effective treatment method for enhancing bowel function in cancer patients.^[13,14]^ Furthermore, most included RCTs suffered from poor quality. Hence, a comprehensive review that could assess the efficacy and safety of acupuncture for enhancing early recovery of bowel function in cancer patients is urgently needed.

## 2 Methods and analysis

The protocol has been registered on PROSPERO 2016 (registration number: CRD42016049633). This protocol adheres to the Preferred Reporting Items for Systematic Reviews and Meta-Analyses Protocols (PRISMA-P) statement guidelines (see File 1 Supplemental Content, which represents the PRISMA-P checklist).^[15]^ We will document the essential protocol amendments in the full review.

### 2.1 Inclusion criteria for this review

#### 2.1.1 Study types

All randomized controlled trials (RCTs) without restrictions on publication status will be included. Nonrandomized studies will be excluded for further data syntheses while the data of acupuncture group will be extracted for safety assessment. Completed trials, trials using parallel design will be included.

#### 2.1.2 Participants

Participants are adults aged 18 years or older who have been formally diagnosed with cancer after surgery. There will be no limitations on gender, education, ethnicity, tumor stage (patients with American Society of Anaesthesiologists grading I–III) and surgical operation.

#### 2.1.3 Interventions

Acupuncture is used as the sole treatment in this study. Related therapies such as manual acupuncture, fire needle and plum blossom needle, dermal needle, acupressure, electroacupuncture will be included. Studies that evaluated auricular acupuncture, laser acupuncture, pharmaco-acupuncture, micro-acupuncture, or acupoint injection will be excluded (the methodology and principles in mechanism differ from the TCM). Control interventions such as no acupuncture, placebo acupuncture, sham acupuncture, and drug therapy will be included. Acupuncture compared with placebo/sham treatment, acupuncture in addition to another intervention versus another intervention alone (the same as the acupuncture group) will be included. RCTs compared acupuncture directly with different types of TCM (e.g., another form of acupuncture, herbal decoction) will be excluded from this study.

#### 2.1.4 Outcome measures

*Primary outcomes* Improvement in bowel function will be assessed by the time to first passing flatus and the time to defecation, measured in hours, from the time the surgery ended until the first observation, or other validated scales.^[16]^

*Secondary outcomes* We will look at 5 main secondary outcomes: time to first bowel sound, visual analog scale (VAS) pain score, postoperative analgesia requirement, duration of hospital stay, risk of POI.^[17]^ In addition, any adverse events related to acupuncture therapy will be measured (if available).

Primary and secondary outcomes based on an unreliable assessment such as “cured,” “improved,” and “failed” will be excluded. Studies that did not report at least 2 of aggregate outcomes will be excluded.

### 2.2 Search methods to identify studies

#### 2.2.1 Electronic searches

A systematic search will be conducted from the time of their inception to January 2017 by using the following databases: Medline, EMBASE, Cochrane CENTRAL, the Cumulative Index to Nursing and Allied Health Literature, Allied and Complementary Medicine Database, the Chinese Biomedical Literature Database, the China National Knowledge Infrastructure, VIP Information, Wanfang Data, 1 Japanese database (Japan Science and Technology Information Aggregator, Electronic), and 2 Korean Medical Databases (Korean Studies Information, and Data Base Periodical Information Academic). The references of included publications will be tracked to identify the possible candidates. The complete manuscripts of all pertinent studies published were retrieved, and restriction will be made to RCTs. There will be no language restrictions. To ensure a broad search, Medical Subject Headings (MeSH) such as RCT, acupuncture, ileus, gastrointestinal function are included. Titles and abstracts will also be searched as well as keywords related to MeSH words. Supplementary searches will be conducted by scanning reference lists of all relevant articles (including conference proceedings, reviews, letters), by manually searching for relevant journals and cross-examining to retrieve additional trials. Additionally, we will manually search OpenGrey.eu for gray literature and databases of ongoing trials (http://www.clinicaltrials.gov; http://www.who.int/ictrp/en/; http://www.google.cn ) to avoid the risk of missing eligible RCTs. The words used in search of Chinese and other databases have the same meaning as those used in the English databases (see File 2 Supplemental Content, which represents the search strategy for Cochrane CENTRAL, and will be modified to suit other databases).

### 2.3 Data evaluation and collection

#### 2.3.1 Study selection

**Figure 1:**
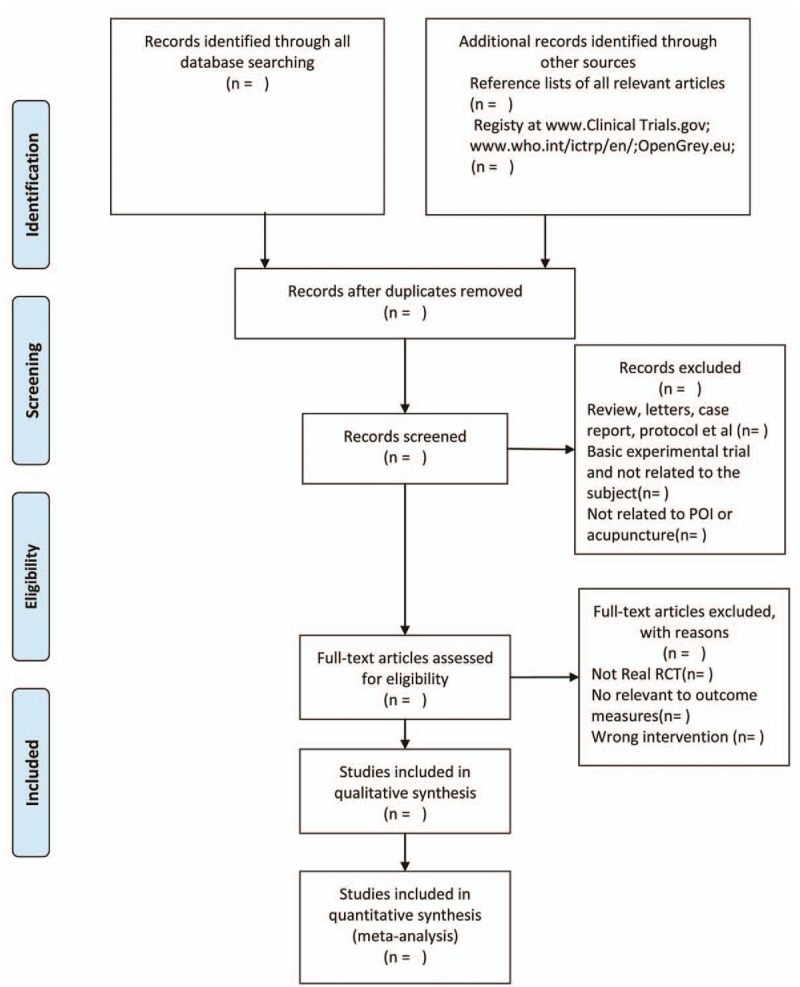
The PRISMA flow chart.

Two trained reviewers (Y-HL and J-BZ) will independently screen both the titles and abstracts to identify all searched studies. After the exclusion of the duplicated and apparently irrelevant studies, a further step will be reviewed in full text and make a decision on whether they meet the predefined inclusion criteria. Any discrepancies were discussed and resolved agreement or consulted a senior researcher (H-SL). The details of selecting process will be presented in the PRISMA flow chart (see Fig. 1).

#### 2.3.2 Data extraction and management

Searched records will be uploaded to ENDNOTE X7 management software, while a predesigned data extraction form will be created. Draft data extraction form will be first used to conduct the pilot tests, and then we will revise it to gain a final form. Two reviewers will review all the records independently, and the relevant information will be extracted using the data extraction form (see File 3 Supplemental Content, which represents the items for data extraction). Excluded studies and exclusive reasons will be listed. Relevant articles will be sorted and cross-examined. We will conduct calibration exercises to ensure consistency across the reviewers. A senior reviewer (H-SL) will be consulted to resolve the discrepancies and uncertainties. When the details were not granted explicitly in an article, the authors will be contacted. The extraction items form will be extracted as followings: patients characteristics: median age, sex ratio, anesthesia type, surgery type, cancer type, and follow-up; basic study characteristics: first author, publication year, type of design, sample size, acupuncture/control type, acupuncture points, treatment course, method of randomization, and allocation concealment and missing data; outcomes: time to first flatus, time to first defecation, time to first bowel sound, postoperative pain, postoperative analgesic requirement, risk of POI, and length of hospital stay. The details of acupuncture treatment was extracted according to the Standards for Reporting Interventions in Controlled Trials of Acupuncture (STRICTA) reporting guidelines, could improve the completeness and transparency of reporting quality in RCTs of acupuncture. A checklist item included needle depth, needle location, name and number of acupuncture points selected, “Deqi” sensation, and duration of treatment sessions ^[18]^ (see File 4 Supplemental Content, which represents the details for acupuncture treatment).

#### 2.3.3 Quality assessment

The reporting quality of included original studies will be assessed by using the Cochrane risk-of-bias (ROB) tool outlined in the Cochrane Handbook for Systematic Reviews of Interventions.^[18,19]^ The completeness of STRICTA checklist will be reviewed independently by two reviewers (Y-HL and J-BZ). The ROB quality items conducted were as follows: random sequence generation (selection bias), allocation concealment (selection bias), blinding of participants and personnel (performance bias), blinding of outcome assessment (detection bias), incomplete outcome data (attrition bias), selective reporting (reporting bias), other bias. After assessing all the domains, the risk of bias will be categorized as a low, high, and unclear risk of bias. For the nonrandomized study, the quality of observational study (including cohort studies) will be assessed with the Newcastle–Ottawa Scale (NOS).^[20]^ A senior reviewer (H-SL) will be consulted to resolve the conflicts.

#### 2.3.4 Measures of treatment effect

We will calculate the effect size for each study and generate an overall effect size after synthesizing. Dichotomous data will be presented as the risk ratio (RR). Mean difference (MD) with 95% CI will be used for effect estimates when outcome measurements are on the same scale while standardized mean difference (SMD) will be used as various scales.

#### 2.3.5 Unit of analysis issue

In this review, we will prefer to include data from parallel design studies. In the case of crossover designed trials, we will use data from the first session. If multiple acupuncture arms exist, we will carry out multiple meta-analyses respectively instead of combining the results. If there are multiple assessment time points, we will focus on an only 1-time point in the analysis.

#### 2.3.6 Dealing with missing data

Once there is missing or incomplete trial data, we will try to request additional information from the corresponding authors of the original trials if possible. If we are unable to obtain the missing data, an intention-to-treat analysis will be performed and tested by a sensitive analysis. Otherwise, we will synthesize the rest of available data. The impact of missing data will be discussed if necessary.

### 2.4 Statistical methods

#### 2.4.1 Assessment of heterogeneity

Either random-effects or fixed-effects model will be utilized in the meta-analysis. We will prefer to use the random-effects model if the heterogeneity between multi-studies and wider intervals is examined. Cochrane *Q* test and Higgins *I*^*2*^ test, which calculate the variant proportion, will be used to assess the statistical heterogeneity. We will calculate the Higgins *I*^*2*^ statistic, a value exceeds the boundary point 50% will be addressed as significant heterogeneity.^[21]^ If a significant value is observed, we will conduct a subgroup analysis to identify the possible courses.

#### 2.4.2 Assessment of reporting biases

If more than 10 trials were included in the meta-analysis, we would rate funnel plot asymmetry by using Begg and Egger tests, and defined *P* value <0.05 as significant publication bias.^[22]^ Moreover, the contour-enhanced funnel plot will be conducted to distinguish publication bias from other biases (if necessary).

#### 2.4.3 Data synthesis

For each trial, the combined estimates will be computed by random-effect models and fixed-effect models. If the *I*^*2*^ statistic is less than 50%, we assume the differences between each trial share equal chance, and the fixed-effects model will be used for data synthesis. If the *I*^*2*^ statistic is higher than 50%, we assume each trial varies markedly, and the random-effects model will be used. Subgroup analysis, meta-regression, or sensitivity analysis will be conducted to explore the potential sources of clinical or methodological heterogeneity. All statistical analyses will be performed using RevMan 5.3 (Cochrane Collaboration) and Stata 12.0 (StataCorp). The results will be calculated as risk ratio (RR) for dichotomous data and standardized mean difference (SMD)/mean deviation (MD) for continuous data. All *P* values were 2-sided. A systematic narrative synthesis will be conducted if it is not possible to complete any meta-analysis.

#### 2.4.4 Subgroup analysis and investigation of heterogeneity

Heterogeneity arising from the acupuncture type (including manual acupuncture, electroacupuncture, fire needle, warm needle), control type (including no acupuncture, sham acupuncture, usual care), and clinical differences will be considered in this review. As confounding factors are not consistent in each trial, potential sources of heterogeneity will be identified by a meta-regression method (at least 10 trials, influence factors such as cancer type, intervention course, treatment frequency, and the geographic region will be detected).

#### 2.4.5 Sensitivity analysis

We will conduct a sensitivity analysis by removing 1 or several studies (high-risk bias or results without STRICTA details) to explore the potential sources of heterogeneity and repeat the meta-analysis to evaluate the impact of excluded trials on the total estimate. In addition, we will assess the sample size and different models to guarantee the robustness of our results.

#### 2.4.6 Grading the quality of evidence

We will use the Grading of Recommendations Assessment, Development and Evaluation (GRADE) approach to describe the overall quality of the outcome.^[23]^ The quality of outcomes will be categorized as high, moderate, low, and very low.

## 3 Discussion

Cancer patients who undergo surgical procedure often suffer from bowel dysfunction and POI.^[11]^ The cause of bowel dysfunction and POI is still unclear. The pathophysiology of POI is considered multifactorial. Factors include the complicated disturbances in immunological, inflammatory, neurological, and receptor-mediated functioning.^[24]^ Pharmacological agents such as cyclooxygenase 2 (COX-2) inhibitors, ghrelin agonists, and opioid agonists always bring side effects, such as cardiovascular adverse events and immunosuppressive effects; laparoscopic technique has been proven to reduce the incidence of POI, while the costs minimize its use ^[10]^. In addition to these treatments, acupuncture has become a promising option for gastrointestinal disease.

Although the exact mechanism of how acupuncture may reduce POI and enhance bowel function is unknown, acupuncture has been shown to improve gastrointestinal dysrhythmia,^[25,26]^ secretion,^[27]^ accelerate solid gastric emptying,^[28]^ and restore impaired gastrointestinal motility mediated via the cholinergic pathway.^[29]^ Extensive research indicates that acupuncture has the potential to treat gastrointestinal disorders by regulating the gastrointestinal barrier, visceral sensitivity, and the brain-gut axis.^[30]^ A recent preclinical study has revealed that electroacupuncture administered at ST36 promotes the recovery time of POI by the exciting nucleus of the solitary tract neurons.^[31]^ Previous RCTs have been performed to investigate the effect of acupuncture in cancer patients with bowel dysfunction or POI. One RCT^[32]^ demonstrated no significant difference of acupuncture compared with sham or no acupuncture in cancer patients with POI, while acknowledging the use of epidural anesthesia might have diminished the possible effects of acupuncture mediated by neural mechanisms. Then, another RCT^[33]^ conducted a larger and more rigorous randomized study, excluded patients who had received anesthesia or analgesia, and minimized the risk of randomization and allocation concealment. They found that electroacupuncture reduced the duration of POI, and opioids consumption, compared with sham or no acupuncture in colorectal cancer. Two previous systematic reviews evaluated the use of acupuncture broadly for cancer care but did not provide any determined result for bowel function and POI in cancer patients.^[13,14]^

In this review, we aim at analyzing all trials and exploring whether acupuncture is associated with the estimated effect of bowel function and POI in cancer. Besides, data of nonrandomized study will be extracted for safety assessment. For an intervention that is effective, low cost, and has few side effects, acupuncture is worth of clinical generalization and application. We hope that the findings of this review will provide important clinical implications.

## Supplementary Material

Supplemental Digital Content

## Supplementary Material

Supplemental Digital Content

## Supplementary Material

Supplemental Digital Content

## Supplementary Material

Supplemental Digital Content
