# Supplementary material for: Acupuncture for enhancing early recovery of bowel function in cancer: Protocol for a systematic review
Source: Medicine (Baltimore). 2017 Apr 28;96(17):e6644. doi: 10.1097/MD.0000000000006644 (PMC5413228; doi:10.1097/MD.0000000000006644)
Supplement: Supplemental Digital Content [file medi-96-e6644-s002.pdf]

Supplemental Digital Content. File 2 represents the search strategy for Cochrane central database.

Search strategy used in CENTRAL database

Number	Search terms
1	MeSH descriptor: [acupuncture] explode all trees
2	MeSH descriptor: [acupuncture therapy] explode all trees
3	Electroacupuncture: ti,ab,kw
4	Acupoint: ti,ab,kw
5	Plum blossom needle: ti,ab,kw
6	Fire needle: ti,ab,kw
7	Dermal needle: ti,ab,kw
8	Electric stimulation therapy: ti,ab,kw
9	Acupressure: ti,ab,kw
10	Acupuncture therapy: ti,ab,kw
11	1 or 2 or 3 or 4 or 5 or 6 or 7 or 8 or 9 or 10
12	MeSH descriptor: [neoplasms] explode all trees
13	Tumor: ti,ab,kw
14	Neoplasia: ti,ab,kw
15	Cancer: ti,ab,kw
16	Benign neoplasm: ti,ab,kw
17	Neoplasm,benign: ti,ab,kw
18	12 or 13 or 14 or 15 or 16 or 17
19	MeSH descriptor: [ileus] explode all trees
20	Gastrointestinal dysfunction: ti,ab,kw
21	Gastrointestinal disorder: ti,ab,kw
22	Paralytic ileus: ti,ab,kw
23	Intestinal Obstruction: ti,ab,kw
24	Pseudo obstruction: ti,ab,kw
25	Postoperative complication*: ti,ab,kw
26	19 or 20 or 21 or 22 or 23 or 24 or 25
27	11 or 18 or 26
