# Supplementary material for: Acupuncture for enhancing early recovery of bowel function in cancer: Protocol for a systematic review
Source: Medicine (Baltimore). 2017 Apr 28;96(17):e6644. doi: 10.1097/MD.0000000000006644 (PMC5413228; doi:10.1097/MD.0000000000006644)
Supplement: Supplemental Digital Content [file medi-96-e6644-s003.docx]

Supplemental Digital Content. File 3 represents the data extraction items.

**Data extraction form items**

| **Category** | **Description** |
| --- | --- |
| **Patients characteristic** |  |
| Median age | Average age and range of included patients |
| Sex ratio | Sample size of gender and ratio |
| Anesthesia type | i.e. epidural anesthesia; general anesthesia |
| Surgery type | i.e. laparoscopic surgery |
| Cancer type | i.e. colorectal cancer; gastric cancer |
| Tumor stage | Number of patients in each stage |
| Follow-up | Period of follow-up |
| **Basic study characteristic** |  |
| First author | Name of the first author |
| Publication year | Published year of included trials |
| Publication region | Published country |
| Type of design | Design of included trials |
| Sample size | Sample size of intervention and control group |
| Acupuncture type | i.e. electroacupuncture |
| Acupuncture points | i.e. ST-36(Zusanli) |
| Treatment course | Intervention details, i.e. intensity and frequency |
| Risk of bias | Details of ROB, i.e. randomization method |
| **Outcome** |  |
| Time to first flatus | Surgery ended time to first passing flatus |
| Time to first defecation | Surgery ended time to first passage of stool |
| Time to first bowel sound | Surgery ended time to first bowel sound |
| Postoperative pain | VAS immediately after 1 week of surgery |
| Postoperative analgesic requirement | Consumption of opioid |
| Length of hospital stay | Time from operation to discharge |
