# Supplementary material for: Acupuncture for enhancing early recovery of bowel function in cancer: Protocol for a systematic review
Source: Medicine (Baltimore). 2017 Apr 28;96(17):e6644. doi: 10.1097/MD.0000000000006644 (PMC5413228; doi:10.1097/MD.0000000000006644)
Supplement: Supplemental Digital Content [file medi-96-e6644-s004.docx]

**Supplemental Digital Content. File 4 represents the details of STRICTA items.**

**Reporting quality of RCTs based on STRICTA**

| Item | Detail | Study 1 | Study 2 | Study 3 | Study 4 | Study5 | Comment |
| --- | --- | --- | --- | --- | --- | --- | --- |
| 1.  Acupuncture rationale | 1a) Style of acupuncture (e.g. Traditional Chinese Medicine，Japanese，Korean，Western medical，etc) |  |  |  |  |  | Style of acupuncture |
|  | 1b) Reasoning for treatment provided， literature sources，and/or consensus methods，with references where appropriate |  |  |  |  |  |  |
|  | 1c) Extent to which treatment was varied |  |  |  |  |  | “Y” is needed to state the detailed description |
| 2.  Details of needling | 2a) Number of needle insertions per subject per session (mean and range where relevant) |  |  |  |  |  | Number of treated  acupuncture per point per session |
|  | 2b) Names (or location if no standard name) of points used (uni/bilateral) |  |  |  |  |  |  |
|  | 2c) Depth of insertion，based on a specified unit of measurement |  |  |  |  |  |  |
|  | 2d) Response sought (e.g. de qi or muscle twitch response) |  |  |  |  |  |  |
|  | 2e) Needle stimulation (e.g. manual，electrical) |  |  |  |  |  | Procedure and technique for acupuncture |
|  | 2f) Needle retention time |  |  |  |  |  | Time of treatment per point |
|  | 2g)Needle type (diameter，length，and manufacturer) |  |  |  |  |  | Detailed feature |
| 3.  Treatment regimen | 3a) Number of treatment sessions |  |  |  |  |  |  |
|  | 3b) Frequency and duration of treatment sessions |  |  |  |  |  |  |
| 4.  Other components of treatment | 4a) Details of other interventions administered to the acupuncture group (e.g. moxibustion，cupping，herbs，exercises) |  |  |  |  |  |  |
|  | 4b) Setting and context of treatment，including instructions to practitioners，and information and explanations to patients |  |  |  |  |  |  |
| 5.  Practitioner background | 5) Description of participating acupuncturists (qualification or professional affiliation，other relevant experience) |  |  |  |  |  |  |
| 6.  Control or comparator interventions | 6a) Rationale for the control or comparator in the context of the research question，with sources that justify this choice |  |  |  |  |  |  |
|  | 6b) Precise description of the control or comparator. If sham acupuncture or any other type of acupuncture-like control is used，provide details as for Items 1 to 3 above. |  |  |  |  |  |  |

*N* not adequately reported; *Y* adequately reported ; *RCTs* randomized controlled trials; *STRICTA* standards for reporting interventions in clinical trials of acupuncture
